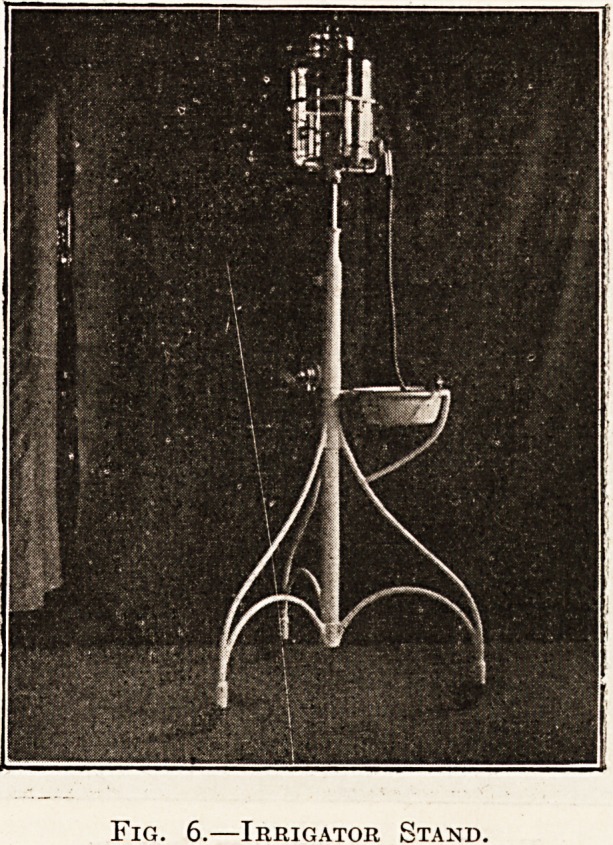# The Units of General Hospital Construction

**Published:** 1907-06-01

**Authors:** 


### HOSPITAL ADMINISTRATION.

### CONSTRUCTION AND ECONOMICS.

### THE UNITS OF GENERAL HOSPITAL CONSTRUCTION.

THE SURGICAL WARD UNIT.—(*Continued.*)

In the construction of some operating theatres unnecessary expense has been incurred in attempting to thin the window-frames, in order to avoid shadows being cast on the table, and in consequence the frames are made of iron. Wood frames

Fig. 3.—An Ambulance Trolley.

are equally suitable and much less costly, and where the building has a northern exposure there is no risk of shadow. The windows and doors should fit closely without projections or ledges, in order that no crevices may be left where dust can lodge. In some cases the doors are made of iron, but a hard wood door with a plain, polished surface, having no panels or mouldings, serves the same purpose. The furnishings should be of the simplest type, to prevent the least possibility of collecting dust, and if movable should be on ball-bearing castors.

Operating Tables.

There is no end to the variety of operating tables, and their cost varies from £10 to £100. The simpler the table, the less there is to keep clean; an excellent table, meeting all the requirements of modern surgery, can be got for about £15. It is always an advantage to have the ambulance trolley of similar dimensions to the operating table. Fig. 3 shows such a trolley. By means of the lever action it is made a fixture resting on two legs, and can be used as a table in emergency, or the third wheel can be put into action and used as a trolley. Fig. 4 shows a simple but most useful stand for instrument trays: the one tray contains the sterilised instruments, and the other, instruments that have been used. Fig. 5 shows receiver for soiled dressings with a cover, avoiding the exposure of blood-stained dressings when they are being removed to the destructor. Fig. 6 shows an irrigator stand on ball bearing castors; there is a lever with a check action at the side for raising the vessel three or four feet in height, and preventing the possibility of the vessel coming down suddenly and getting smashed.

Adjoining the operating theatre are the anæsthetic room and the robing room for surgeon and assistants on the one side; on the other are the sterilising and surgical dressings room. The doors to these are placed under the side galleries. None of those rooms open directly to the main corridor, and the risk of dust blowing into the theatre or its annexes is thus obviated. The students enter the galleries from stone stairs and have no direct communication with the area of the theatre. Those under the window enter through the galleries and are cut off from the operating area by a terrazzo wall three feet six inches in height.

The Lecture Room.

If the lecture room is fitted with benches and tables, it can be utilised as a general class room for microscopical demonstrations or other purposes. Arrangements should be made whereby the lecturer may give a lantern demonstration and show stereoscopic photographs. The room should therefore be fitted with dark blinds, in order that it may be readily darkened. These blinds should have a spring action, and be rolled up into boxes with close-

Fig. 4.—The "Mackintosh" Instrument Stand.

fitting lids, so that when not in use they are free from dust. In addition, a heavy electric wire sufficient to carry 20 amperes of current must be led to the room, and a plug and switch fixed in a suitable position. A lantern screen should also be provided.

There remain the special units such as those for diseases of the ear, diseases of the throat, diseases of the skin, gynæcology, burns, and septic cases unsuitable for the general wards. In all of those, with the exception of the gynæcological, provision must be made for both male and female patients. The gynæcological unit should always be as far removed as possible from the septic, and even the burn and skin wards. These units are simply modifications of those already described, and should not be inferior to them either as regards cubic capacity or constructive detail.

### Lighting Arrangements.

In the special units for the treatment of diseases of the ear, nose, and throat a room should be provided where patients can be examined by artificial light, arrangements being made whereby the room can be readily darkened. Some specialists prefer to have this room fitted up with electric light only. In this case provision must be made for the heating of the laryngoscopic mirrors, either by hot water or by an electric heater. Other specialists prefer to use gas; an ordinary argand burner is found quite serviceable, and serves the double purpose of illumination and also for heating the mirrors.

In the unit for the treatment of diseases of the skin a few special bath fittings must be arranged for. Besides two sitz baths, a hot-air bath should be supplied. This hot-air bath can be arranged in the form of a cabinet, heated by a number of incandescent electric lamps placed in front of reflectors. It has the advantage that it can be placed in an ordinary, well-ventilated room, so that while the patient is in the bath he is breathing fresh, pure air. Various forms of these electric baths have been devised. In some the patient can have the bath in the recumbent posture, which is a decided advantage. In others the patient sits upright, his head only showing outside the cabinet.

Fig. 5.—Receiver for Soiled Dressings.

### An Isolation Room.

Adjoining the skin wards a room should be provided for the isolation and treatment of diseases of the skin, such as scabies, etc. The linen and other articles used in this room must be kept separate and distinct; the linen should be specially marked, so that it may be thoroughly disinfected and washed in order to avoid the possibility of it getting mixed with the other linen of the hospital.

### The Gynæcological Room.

In the gynæcological unit, although only one large ward is required, a side ward large enough to accommodate three or four patients should adjoin the ward, so that patients who have undergone serious abdominal operations may be kept perfectly quiet for at least the first 24 hours after operation.

Fig. 6.—Irrigator Stand.

The unit for septic cases should either be designed as a separate building or be placed as far as possible from any other surgical ward. It is necessary to provide a small operating room for these septic cases, but no accommodation for students need be made.

### The Treatment of Burns.

Special provision should be made for burns in order that severe cases may be treated in water baths. These latter should be supplied with warm water and an arrangement made whereby this warm water can be mixed with cold according to requirements. A constant flow must be maintained to ensure its purity. The patient is suspended in the water in a special form of hammock. Air or water cushions may be utilised to add to his comfort. The bath should be emptied and thoroughly cleaned out every day, and this can be done without removing the patient. By means of this special arrangement severe burns of the buttocks, back, and thighs can remain in the bath practically undisturbed for weeks or even months. It is important that the water be regularly changed without allowing the temperature of the bath to drop below 99° to 100° F. This is an expensive form of treatment, as constant supervision is required, but when a hospital is situated in a district where severe burns, such as from molten metal, are frequently admitted, the expense is justifiable.

## Figures and Tables

**Fig. 3. f1:**
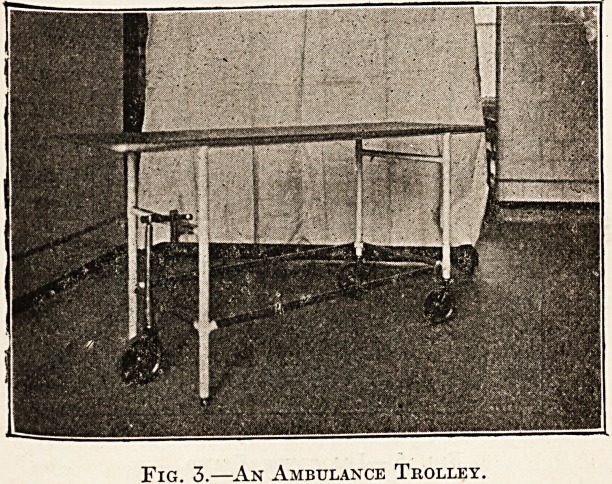


**Fig. 4. f2:**
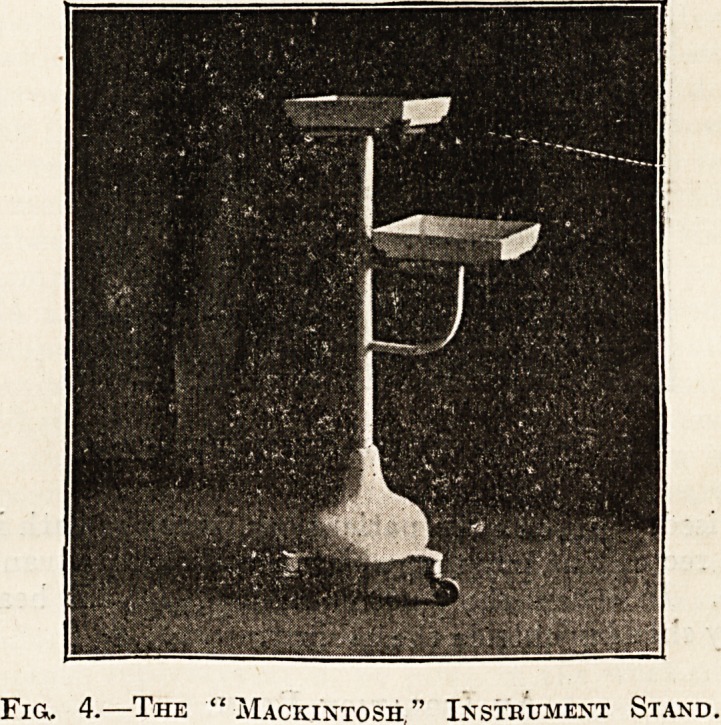


**Fig. 5. f3:**
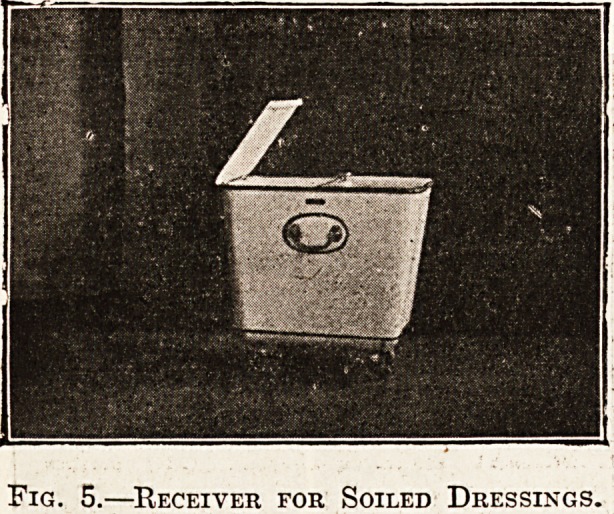


**Fig. 6. f4:**